# Analgesic Effect of the Topical Use of Dexamethasone in Ultrasound-Guided Axillary Brachial Plexus Blockade: A Prospective, Randomized, Double-Blind, Placebo-Controlled Study

**DOI:** 10.7759/cureus.12971

**Published:** 2021-01-28

**Authors:** Aikaterini Chazapi, Panagiotis Lepetsos, Zoe Gambopoulou, Ioanna Siafaka, Erifylli Argyra, Athina Vadalouka

**Affiliations:** 1 Department of Anaesthesiology, KAT Hospital, Kifissia, GRC; 2 4th Department of Orthopaedics, KAT Hospital, Kifissia, GRC; 3 1st Department of Anaesthesiology, University of Athens Medical School, Athens, GRC

**Keywords:** dexamethasone, ropivacaine, axillary block, brachial plexus

## Abstract

Introduction

Increasing the duration of regional anesthesia in orthopedic surgery is of vital importance, as it prolongs postoperative analgesia, allowing faster rehabilitation of patients. Dexamethasone has been found to extend the block duration in animal and human studies. The aim of this study is the assessment of the effect of the addition of dexamethasone to ropivacaine on the onset and duration of axillary brachial plexus block, along with the intensity of postoperative pain.

Methods

Forty patients undergoing below-elbow surgery under ultrasound-guided axillary brachial plexus block were randomly allocated to receive either 30 mL ropivacaine 0.75% with 2 mL of saline (Group A, n = 20) or 30 mL ropivacaine 0.75% with 2 mL of dexamethasone (4 mg) (Group B, n = 20). Sensory and motor blockade were assessed, with the use of the pinprick test and the modified Bromage scale, at five, 10, 15, and 20 min after the block. The duration of analgesia, intensity of postoperative pain, postoperative opioid consumption, overall satisfaction, and perioperative complications were compared between the two groups.

Results

We found no difference at the mean onset time of the sensory and motor block between the two groups. The mean duration of postoperative analgesia was three hours higher in the dexamethasone group (15.85 ± 4.82 versus 11.75 ± 6.81, p-value = 0.035). Pain intensity was lower in the dexamethasone group, at six and 12 hours after surgery (3.45 ± 1.79 versus 4.65 ± 1.79, p-value = 0.040). Postoperative opioid consumption, patient overall satisfaction, and perioperative complications were not significantly different between groups.

Conclusions

Dexamethasone prolongs the duration of ropivacaine in an axillary brachial plexus block and decreases postoperative pain in patients subjected to below-elbow surgery.

## Introduction

As upper limb operations are associated with severe postoperative pain, it is important to apply techniques with a more powerful analgesic effect, without the use of intravenous (IV) analgesics and opioids that have significant side effects. An axillary brachial plexus block has gained more popularity for the limitation of postoperative pain for forearm and wrist surgeries is a reliable and safe option, well-tolerated by patients, with a low complication rate [1]. However, even with the use of long-lasting local anesthetics, peripheral blockades will reliably provide a limited duration of analgesia [2].

Increasing the duration of regional anesthesia in orthopedic surgery is of vital importance, as it prolongs postoperative analgesia, allowing faster rehabilitation of patients [3]. Ropivacaine is widely used in an axillary brachial plexus block for its extended action. Ultrasound can improve the precision of the blockade or reduce the block performance time [4]. However, it is not feasible to extend the duration of the blockade by increasing the dose of local anesthetic. For this reason, various additives have been used in the local anesthetic solution in order to ensure the prolongation of analgesia. These additives include epinephrine, α2 agonists, such as clonidine and dexmedetomidine, midazolam, and dexamethasone, which either appear to cause local vasoconstriction or act directly on peripheral nerves through anti-inflammatory action [5].

Several studies have shown the analgesic effect of local and systemic corticosteroids when added to bupivacaine [6]. Dexamethasone is a systemic glucocorticoid commonly used to decrease postoperative pain, nausea, and vomiting and to improve the postoperative quality of recovery [7]. Recently, data in the literature have reported its use for the extension of the analgesic duration of peripheral nerve blocks and especially in brachial plexus blockades [8]. The effect of dexamethasone on block duration may vary, depending on anesthetic factors such as the type of block, the local anesthetic used, the dose of dexamethasone, and the route of administration (topical or intravenous) [9]. In addition, the safety of perineural dexamethasone also needs to be further examined, after the observation of crystal formation when several local anesthetics, including ropivacaine, are combined with dexamethasone [10].

The aim of this study is the assessment of the effect of the addition of dexamethasone to ropivacaine on the onset and duration of the axillary brachial plexus block, along with the intensity of postoperative pain.

## Materials and methods

**Figure 1:**
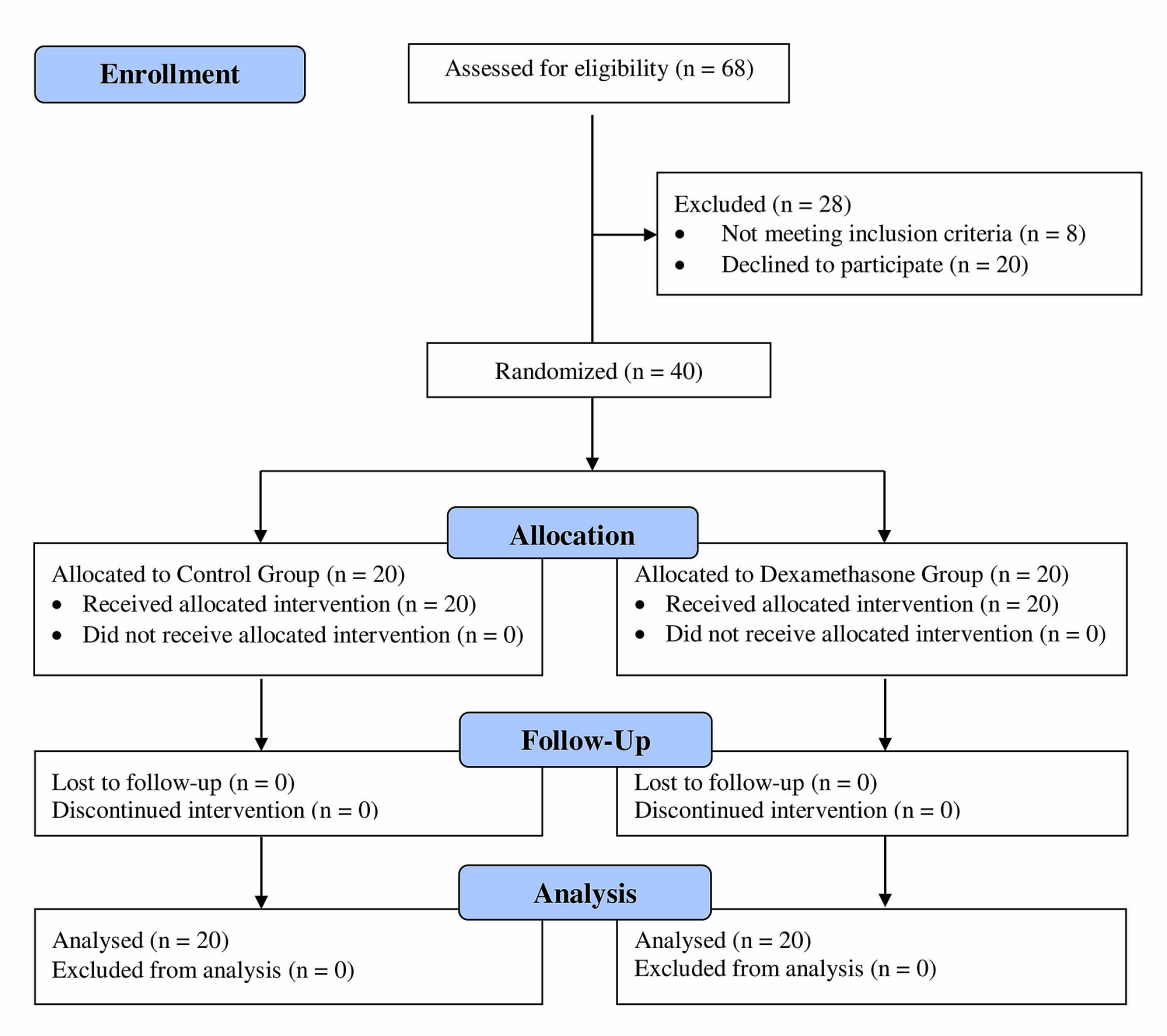
Flow chart of patients included in the study according to the CONSORT statement CONSORT: Consolidated Standards of Reporting Trials

This was a prospective, randomized, double-blind, placebo-controlled study on patients undergoing ultrasound-guided axillary brachial plexus blockade for below-elbow surgery. After institutional review board approval of the study, all individuals were fully informed of its purpose and signed the relevant consent form. The Consolidated Standards of Reporting Trials (CONSORT) recommendations for reporting the randomized, placebo-controlled clinical trials were followed (Figure 1).

Inclusion criteria were patients aged between 18 and 70 years old, subjected to hand, wrist, forearm, and elbow surgery under axillary brachial plexus blockade. Patients with American Society of Anesthesiologists (ASA) score > III, neurological deficits, allergy in local anesthetics, INR > 1.4, a contradiction in dexamethasone use, diabetes mellitus, or chronic opioid use were excluded from the study.

Patients who met the inclusion criteria in the study were randomized into two groups: group A (control group, n = 20) and group B (dexamethasone group, n = 20). On arrival to the operating room, heart rate (HR), systolic and diastolic arterial blood pressure (SAP/DAP), and peripheral oxygen saturation (SpO2%) were monitored. Ten minutes before the axillary blockade, all patients received 40 mg omeprazole intravenously, 4 mg ondansetron, and a midazolam solution of 0.01 - 0.1 mg/kg for mild sedation.

The patient was placed in a supine position, with the arm in 90° abduction and the elbow in 90° flexion, with the shoulder in external rotation and the whole arm next to the patient's head. An ultrasound device (Sonoscape 2, SonoScape Medical Corp, China) was used to perform the blockade. Initially, the ultrasound probe was placed perpendicular to the axis of the axillary artery, between the biceps and triceps muscles. Once 10 ml of local anesthetic was administered dorsally to the axillary artery, the needle was redirected to the median and ulnar nerve, where another 12-15 ml was administered. Finally, the needle was withdrawn back into the biceps and was redirected to the musculocutaneous nerve. As soon as the needle was adjacent to the nerve, 5-7 ml of local anesthetic was administered.

Group A (control group) received 30 ml (0.75%) of ropivacaine mixed with 2 ml (0.9%) of normal saline (placebo) while group B received 30 ml (0.75%) of ropivacaine mixed with 2 ml (4 mg) dexamethasone. The administered local anesthetic solutions were always prepared by the same anesthesiologist who was the only one who knew their contents while the blockade was performed by a second anesthesiologist unaware of their contents. The type of solution administered was also unknown to the patient.

The pinprick test was used to assess sensory blockade in the area of distribution of the musculocutaneous nerve (forearm), radial nerve (dorsal 1st and 2nd intermetacarpal are), median nerve (palmar side of the tip of the 3rd finger), and ulnar nerve (palmar side of the tip of the 5th finger). The motor blockade was evaluated with the use of the Modified Bromage Scale (MBS) in the radial nerve (thumb abduction), ulnar nerve (thumb adduction), median nerve (thumb opposition), and musculocutaneous nerve (elbow flexion and forearm pronation). The degree of sensory and motor blockade of the axillary block was monitored and recorded by the anesthesiologist performing the blockade 0, 5, 10, 15, and 20 minutes after its execution. Time = 0 was considered as the moment of completion of the technique. Patients who showed a degree of sensory blockade < 1 in 20 minutes after the blockade, were excluded from the study. The onset time of sensory blockade was defined as the time period between the end of injection and loss of pain around the injury site. The onset time of the motor blockade was defined as the time period between the end of injection and complete motor paralysis. Neither the patient nor the anesthesiologist who assessed the sensory and motor blockades was aware of the used anesthetic solution.

The patient was then transported to the operating room where standard monitoring was maintained (electrocardiography, pulse oximetry, and automated sphygmomanometry) and oxygen was delivered through a simple oxygen mask at a rate of 5 L/min. If at the start of the operation, the patient complained of pain (visual analog scale (VAS) > 4) or discomfort in the operated limb, 50 μg of fentanyl was administered intravenously. If pain persisted and surgery could not be continued, the blockade was considered as failed and general anesthesia was added. The patient was then excluded from the study. In addition, any side effects were recorded up to the induction of general anesthesia (nausea, vomiting, SpO2 < 95%, bradycardia, hypotension, etc.). Postoperatively, the patient was taken to the resuscitation unit. Instructions for initial postoperative analgesia consisted of 1 gr of intravenous paracetamol at the time when the patient first complaint of pain (VAS > 4). If the pain persisted, intravenous tramadol was administered. All patients were kept in the hospital overnight.

The following data were recorded and compared between the two groups: 1. Demographic characteristics of the patients (age, gender, weight, height, body mass index (BMI)); 2. Surgical data (type, site, and duration of surgery, surgery on dominant hand); 3. Block performance time, defined as the time period from the first contact of the ultrasound probe with the patient until the end of local anesthetic injection; 4. Time of onset of sensory and motor block; 5. The Ramsay Sedation Scale of the patient at the beginning of the blockade and when the patient entered the post-anesthesia care unit; 6. The side effects that the patient experienced throughout the blockade, the operation, and when he entered the post-anesthesia room; 7. HR, SAP, DAP, and SpO­2 were measured at 0, 5, 10, 15, 30, and 60 minutes after the blockade; 8. Pain intensity was measured with the VAS, graded from 0 (no pain) to 10 (worst experienced pain), at 6, 12, 24, and 48 hours after the start of the blockade both at rest and during passive movement of the joint by the anesthesiologist; 9. The duration of analgesia, defined as the period from drug injection to the first occasion when the patient first complains of pain (VAS ≥ 4); 10. The total number of opioid consumptions requested by the patient in the first 48 hours postoperatively; 11. The degree of patient satisfaction with the quality of postoperative analgesia with the help of the Likert scale.

Statistical analysis

Based on a priori power analysis, it was estimated that at least 16 patients per group were needed to detect a 35% change in the duration and strength of analgesia to achieve a statistical power of 80% at significance 0.05. The baseline characteristics for categorical variables were expressed in number and percentage. The continuous variables were expressed as mean ± standard deviation (SD). Data were evaluated with the Kolmogorov-Smirnov and Shapiro Wilk normality tests. The student’s unpaired t-test was used to compare continuous values with normal distribution. If the distribution of the continuous values was not normal, the Mann-Whitney test was used. Fischer’s exact test was used to compare categorical values. Statistical analyses were performed using PASW 18.0 (SPSS release 18.0; SPSS Inc., Chicago, Illinois) with a level of significance set at p < 0.05.

## Results

Forty patients were included in the study and randomly assigned to the treatment groups. The patient characteristics of the study population are summarized in Table 1.

**Table 1:** Characteristics of the study population BMI: body mass index

	Group Α	Group Β	Total	p-value
	N (%)	N (%)	N (%)	
Gender				0.72
Men	14 (70.0)	16 (80.0)	30 (75.0)	
Women	6 (30.0)	4 (20.0)	10 (25.0)	
Previous operation	3 (15.0)	4 (20.0)	7 (17.5)	1,00
Operation in dominant limb	12 (60,0)	15 (75,0)	27 (67,5)	0,50
Type of operation				1.00
Bone	13 (65.0)	14 (70.0)	27 (67.5)	
Soft tissue	7 (35.0)	6 (30.0)	13 (32.5)	
Site of operation				0.34
Elbow	4 (20.0)	7 (35.0)	11 (27.5)	
Forearm	1 (5.0)	0 (0.0)	1 (2.5)	
Wrist	10 (50.0)	9 (45.0)	19 (47.5)	
Hand	5 (25.0)	4 (20.0)	9 (22.5)	
	Mean (SD)	Mean (SD)	Mean (SD)	p-value
Age (years)	42,1 (13,7)	37,6 (15,3)	39,8 (14,5)	0,52
Weight (kg)	75,6 (16.1)	79,4 (10,5)	77,5 (13,6)	0,38
Height (cm)	173,5 (9,0)	174,2 (7,0)	173,8 (7,9)	0,79
BMI (kg/m^2^)	24,9 (3,9)	26,2 (3,0)	25,5 (3,5)	0,27
Duration of operation (min)	67,8 (23,8)	56,8 (17,9)	62,2 (21,5)	0,11
Duration of block performance (min)	10.50 (2.76)	10.10 (3.01)	10.30 (2.86)	0.66

Group A (control group) included 20 patients (14 men, 6 women), with a mean age of 42.1 ± 13.7 years (range 16 - 69 years). Group B (dexamethasone group) included 20 patients (16 men, 4 women), with a mean age of 37.6 ± 15.3 years (range 18 - 66 years). As shown in Table 1, there was no significant difference associated with the age, gender, weight, height, body mass index (BMI), previous operation, site of operation, type of operation, duration of operation, and duration of block performance between the two groups.

 As shown in Table 2, there were no significant differences in mean onset time of sensory and motor block (p = 0.439 and p = 0.517, respectively), postoperative opioid consumption (p = 0.582). The duration of analgesia was higher in group B (p = 0.035). There was no significant difference in patient sedation at the beginning of the blockade and when the patient entered the post-anesthesia care unit.

**Table 2:** Differences in the onset time of sensory and motor block, duration of analgesia, and postoperative opioid consumption between group A and group B

	Group Α	Group Β	p-value
	Mean (SD)	Mean (SD)	
Onset time of sensory block (min)	18,95 (2,68)	19,50 (1,54)	0,439
Onset time of motor block (min)	19,33 (1,76)	18,89 (2,14)	0,517
Duration of analgesia (hours)	11.75 (6.81)	15.85 (4.82)	0.035
Postoperative opioid consumption (mg)	75.00 (63.87)	65.00 (48.94)	0.582

**Figure 2:**
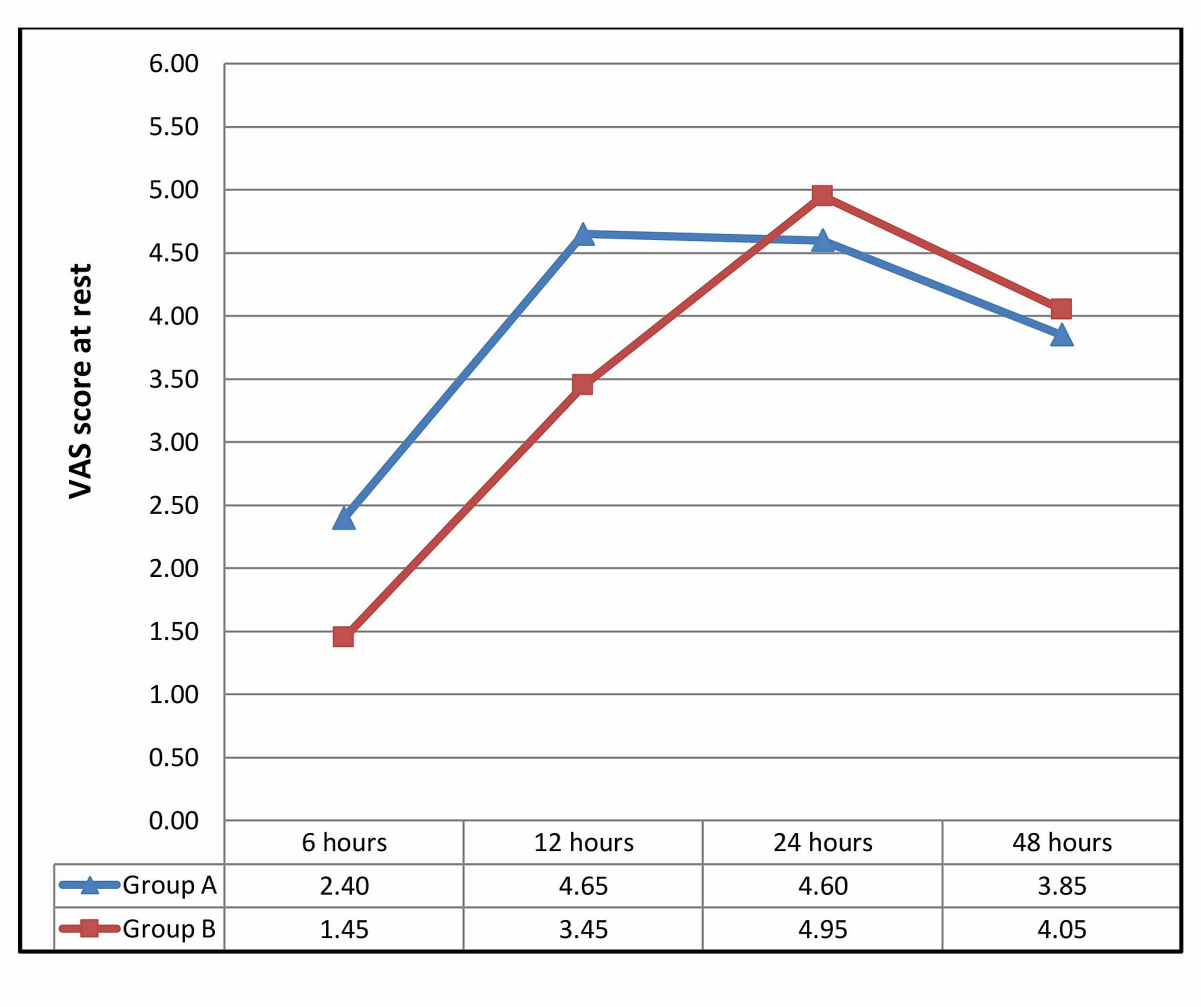
Mean VAS score at rest between the two groups, at 6, 12, 24, and 48 hours after the operation VAS: visual analog scale

**Figure 3:**
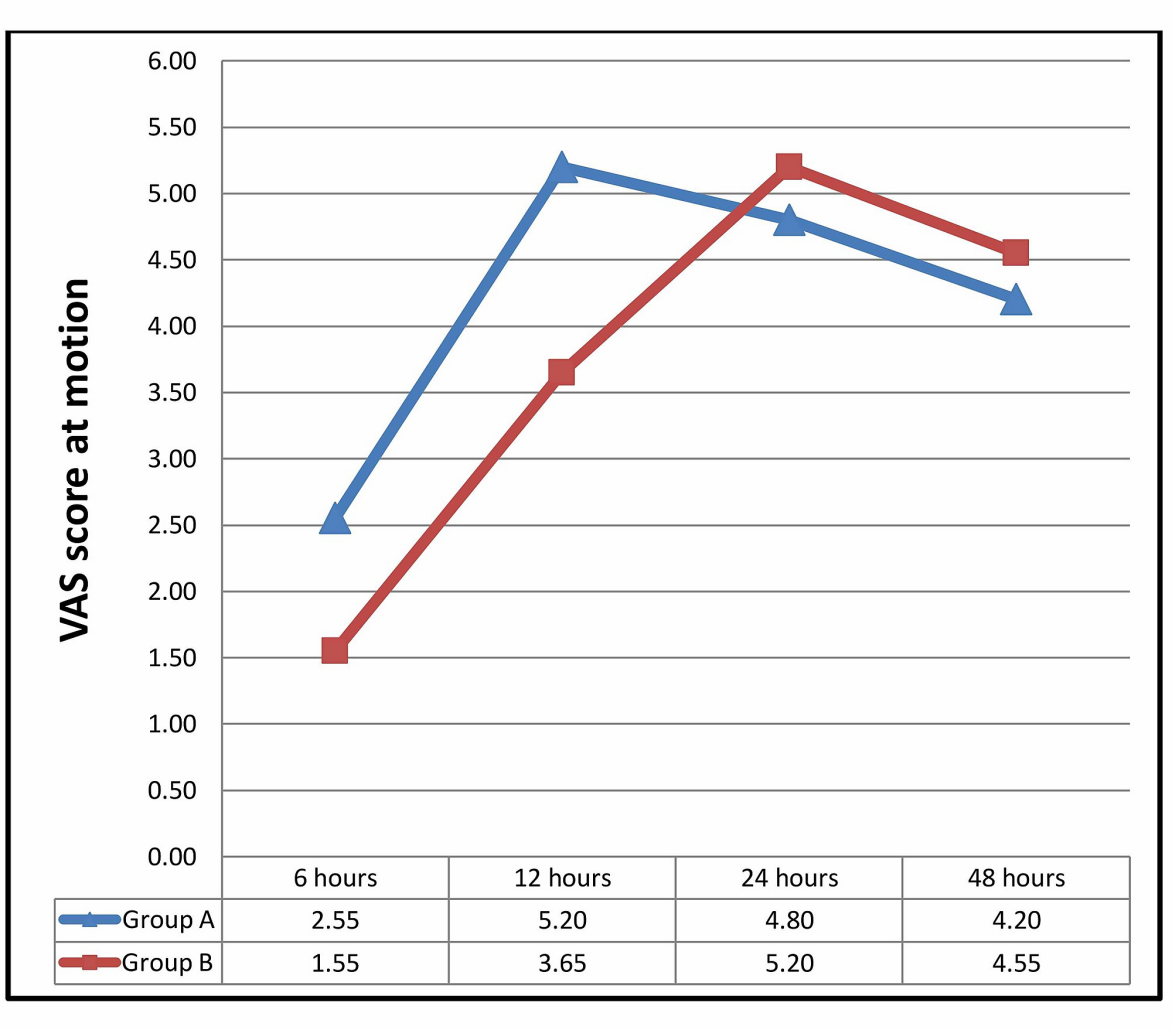
Mean VAS score at motion between the 2 groups, at 6, 12, 24, and 48 hours after the operation VAS: visual analog scale

Table 3 shows the mean VAS score of the patients at 6, 12, 24, and 48 hours after the operation (both at rest or motion of the operated limb). The mean VAS score was significantly lower in group B, at six and 12 hours after surgery. However, there was no significant difference in pain intensity at 24 and 48 hours postoperatively (Figures 2-3).

**Table 3:** Differences in VAS score (at rest or motion) at 6, 12, 24, and 48 hours after surgery between group A and group B VAS: visual analog scale

	Group Α	Group Β	p-value
	Mean (SD)	Mean (SD)	
6 hours (rest)	2,40 (1,31)	1,45 (1,43)	0,035
6 hours (motion)	2,55 (1,23)	1,55 (1,43)	0,023
12 hours (rest)	4,65 (1,79)	3,45 (1,79)	0,040
12 hours (motion)	5,20 (1,85)	3,65 (1,95)	0,014
24 hours (rest)	4,60 (1,31)	4,95 (1,50)	0,438
24 hours (motion)	4,80 (1,24)	5,20 (1,24)	0,314
48 hours (rest)	3,85 (0,67)	4,05 (0,69)	0,357
48 hours (motion)	4,20 (0,83)	4,55 (0,99)	0,236

Table 4 shows the mean changes of hemodynamic variables during surgery. No significant differences were observed in the mean changes of HR, SAP, DAP, or SpO2 during the first 60 minutes of operation between the two groups. No cases of tachycardia or bradycardia, hypertension or hypotension, decrease of SpO2 < 97%, and nausea or vomiting were observed in the study population. There was no difference in overall patient satisfaction with the quality of postoperative analgesia between the two groups.

**Table 4:** Differences in the mean changes of HR, SAP, DAP, and SpO2, at the first 60 minutes of the operation between group A and group B ΔHR: Mean change of HR from 0 to 60 minutes after the operation. ΔSAP: Mean change of SAP from 0 to 60 minutes after the operation. ΔDAP: Mean change of DAP from 0 to 60 minutes after the operation. ΔSpO2: Mean change of SpO2 from 0 to 60 minutes after the operation. HR: heart rate; SAP: systolic arterial blood pressure; DAP: diastolic arterial blood pressure; SpO2: peripheral oxygen saturation

	Group Α	Group Β	p-value
	Mean (SD)	Mean (SD)	
ΔHR (beats/min)	-1.25 (5.00)	0.75 (2.34)	0.117
ΔSAP (mmHg)	1,67 (4,50)	1,25 (3,11)	0.779
ΔDAP (mmHg)	-0.67 (7.04)	0.83 (7.93)	0.613
ΔSpO_2_ (%)	-0.35 (0.59)	0.00 (0.73)	0.102

## Discussion

To the best of our knowledge, this is the first prospective, randomized, double-blind, placebo-controlled study that has shown that the addition of 4 mg of dexamethasone to ropivacaine provides a longer duration of analgesia as compared to ropivacaine alone using an ultrasound-guided axillary block in patients with below-elbow surgery. Furthermore, dexamethasone reduces postoperative pain intensity until 12 hours after the operation.

Several studies in the literature have indicated that the addition of 8 mg dexamethasone to local anesthetic solutions prolongs peripheral nerve block analgesia [11-12]. It has been reported that adding dexamethasone to local anesthetics for a supraclavicular brachial plexus block extends the duration of analgesia with no adverse events [12]. Another randomized controlled trial has also noticed that adding dexamethasone to bupivacaine prolongs the analgesia of supraclavicular brachial plexus block, without any further assessment of pain intensity and the dose of postoperative analgesics [13]. On the other hand, animal studies have reported conflicting results about the analgesic effects of dexamethasone in sciatic nerve blocks [14].

Dexamethasone has been extensively tested in the axillary block with different doses and topical anesthetics. A paper published in 2005 that evaluated the addition of dexamethasone to lidocaine for an axillary brachial plexus blockade arrived at similar conclusions to our study on the mean onset time of sensory and motor block but no data regarding the pain intensity and postoperative use of analgesics were presented [15]. A study published in 2016 evaluated the topical administration of 10 mg of dexamethasone in combination with ropivacaine for axillary brachial plexus blockades. In agreement with our results, the authors found no difference in the mean onset time of the sensory block. However, the authors did not assess motor block, postoperative pain, and duration of analgesia [16]. Similarly, another study concluded that the addition of 8 mg of dexamethasone to prilocaine prolonged the duration of sensory and motor block in patients with axillary brachial plexus blockades [17]. A recent study concluded that the addition of dexamethasone may improve the duration of the axillary block in patients undergoing below-elbow surgery [18].

The addition of a single dose of 8 mg of the intravenous administration of dexamethasone to ropivacaine has been found to delay the onset of postoperative pain after an axillary brachial plexus blockade [19]. However, in an axillary blockade, the perineural administration of 8 mg of dexamethasone in addition to lidocaine, bupivacaine, and epinephrine has been found to provide a longer duration of analgesia in comparison to the intravenous route [9]. Recent data comparing the topical and intravenous administration of dexamethasone in several types of regional anesthesia reported controversial results, showing either greater or equivalent efficacy of the topical versus intravenous route of administration [20].

The effect of dexamethasone in increasing the duration of peripheral nerve blocks can be explained by several theories while the mechanism of the steroid-induced analgesia is not elucidated. On topical application, steroids may produce a degree of vasoconstriction that may decrease the absorption of local anesthetics [11,21]. This steroid-induced vasoconstriction is regulated by the occupancy of classical glucocorticoid receptors, as steroids connect to intracellular receptors and affect nuclear transcription [22]. Moreover, along with its immunosuppressive action, dexamethasone may inhibit potassium channels on pain sensory nerves, blocking pain transmission [11].

The administration of dexamethasone in a nerve sheath is not an absolute indication of this drug, raising some safety concerns [23]. In animal studies, repeated intrathecal injections of small-dose steroids were not correlated with spinal neurotoxicity [24]. Dexamethasone rarely causes nerve injury and, when it does, it usually occurs in the context of needle trauma [25]. However, in the present study, the probability of needle trauma was low, as the ultrasound permitted direct visualization during the performance of the block. In general, single doses and short-term use of dexamethasone is safe to be administered [26]. Nevertheless, the addition of steroids to local anesthetics may not be an indication for diabetic patients, as it can cause hyperglycemia and immunosuppression, increasing the possibility of severe infections [27]. In our opinion, the possibility of immunosuppression induced by a single dose of local corticosteroid cannot be omitted. We chose not to include diabetic patients in our study and we excluded patients with ASA score > III as a precaution for the potential systematic adverse events of dexamethasone. Moreover, dexamethasone did not cause any significant change in the hemodynamic parameters of the patients as well as SpO2 or any other complications, suggesting that it is a safe adjuvant when administered perineurally in axillary blockades.

The optimal dose of perineural dexamethasone remains unclear. In the present study, the dose of the administered dexamethasone was 4 mg. The most common dose for a brachial plexus blockade is 8 mg. For brachial plexus blocks, 4 mg of perineural dexamethasone has provided a longer sensory and motor block and analgesia than intravenous administration [28]. On the other hand, a dexamethasone dose of 8-10 mg provides similar block durations either topically or intravenously [17]. It is possible that lower doses of dexamethasone provide similar results to the effects of postoperative analgesia in comparison to the most commonly used dose of 8 mg dexamethasone. In fact, a recent study showed that even lower doses, such as 1 mg, may provide similar effects during analgesia with higher doses of dexamethasone [29]. We chose to administer only 4 mg of dexamethasone, as current literature suggests a reduction of the volume of local anesthetics used for ultrasound-guided upper extremity blockades [30]. Also, the local and not the systemic administration of dexamethasone was chosen in order to avoid the side effects of systemic glucocorticoids.

In the present study, we chose to use ultrasound-guided blockade for better precision of nerve localization. Ultrasound guidance of peripheral nerve blocks may minimize discomfort by avoiding nerve stimulation and may reduce the number of needle passes [30]. Moreover, we chose to use ropivacaine, as it is a long-acting local anesthetic. Because of its unique phar­macologic properties and fewer adverse events, ropivacaine has been accepted by an increas­ing number of anesthesiologists for peripheral nerve blocks.

It is interesting to note that topical administration of dexamethasone has no effect on late postoperative pain after 24 hours. This finding is in line with a recent meta-analysis by De Oliveira et al. [23]. This may be attributed to the fact that the biological half-life of dexamethasone in plasma is 190 min so 12 hours after its administration, much of its bioavailability has been diminished. Interestingly, no difference was observed in postoperative opioid consumption and overall patient satisfaction from the anesthesia procedure. This may be attributed to the fact that dexamethasone did not affect total pain intensity but only in the first 12 hours after surgery, so after this time, patients may have needed opioid administration for better control of postoperative pain.

Our study does present some limitations. First, the conclusions of the study are specific to the ropivacaine solution that was used. More trials are required for other local anesthetic solutions. Second, the dose of dexamethasone we used was the same (4 mg) and not adjusted to the weight of the patient. Moreover, we did not engage another group with intravenous administration of the same dose of dexamethasone, so we cannot exclude the possibility that prolongation of analgesia occurred because of the systemic effects of dexamethasone.

## Conclusions

The present study has proven that, in an ultrasound-guided axillary brachial plexus block, the topical addition of dexamethasone to ropivacaine significantly prolongs analgesia and decreases the intensity of postoperative pain in comparison to the group given ropivacaine alone. Unexpectedly, there was no difference in postoperative opioid administration for patients receiving dexamethasone. Moreover, the mean time for the onset of sensory and motor blocks along with the hemodynamic changes was similar to those observed for the ropivacaine group. The increase of postoperative analgesia can offer substantial benefits for outpatient upper limb surgery. Taking into consideration the beneficial effect produced by the addition of dexamethasone to ropivacaine, we suggest that the combination of topical dexamethasone/ropivacaine may be used in ultrasound-guided axillary brachial plexus blockade.
